# Neuromuscular and Performance Responses to Resisted Sprint Loads in Elite Female Sprinters

**DOI:** 10.3390/sports13090327

**Published:** 2025-09-15

**Authors:** Mieszko Bartosz-Jeffries, Irineu Loturco, Adam Zając, Adam Maszczyk, Tomás T. Freitas, Pedro E. Alcaraz, Lucas A. Pereira, Artur Gołaś

**Affiliations:** 1 The Institute of Sport Sciences, The Jerzy Kukuczka Academy of Physical Education in Katowice, 40-959 Katowice, Poland; mieszko.bartosz1@gmail.com (M.B.-J.); a.zajac@awf.katowice.pl (A.Z.); a.maszczyk@awf.katowice.pl (A.M.); a.golas@awf.katowice.pl (A.G.); 2 NAR-Nucleus of High Performance in Sport, São Paulo 04753-060, Brazil; lucasa_pereira@outlook.com; 3 Department of Human Movement Sciences, Federal University of São Paulo, São Paulo 04021-001, Brazil; 4 UCAM Research Center for High Performance Sport, UCAM Universidad Católica de Murcia, 30107 Murcia, Spain; tfreitas@ucam.edu (T.T.F.); palcaraz@ucam.edu (P.E.A.); 5 Facultad de Deporte, UCAM Universidad Católica de Murcia, 30107 Murcia, Spain

**Keywords:** resisted sprinting, sprint speed, athletic performance, track and field, elite athletes

## Abstract

This study examined the effects of motorized resisted sprint training (RST) on neuromuscular activation and sprint performance in elite female sprinters. Ten highly trained athletes (age: 23 ± 2.8 years; body mass: 58.3 ± 4.7 kg) performed two maximal 30 m unresisted sprints and six resisted sprints under three different load conditions (i.e., 5%, 10%, and 15% of body mass [BM]), randomized in a counterbalanced design. Surface electromyography (EMG) of eight lower-limb muscles was recorded bilaterally using wearable EMG-integrated shorts. Sprint times were captured using dual-beam photocells, and motorized resistance was applied with the SPRINT 1080 device. Repeated-measures ANOVA revealed a significant load-dependent effect on sprint time (*p* < 0.001, η^2^ = 0.926), with performance decreasing as resistance increased. However, no significant changes were observed in most muscle groups across load conditions, except for a non-significant trend toward increased left gluteus maximus activity (*p* = 0.053, η^2^ = 0.136). Interestingly, greater inter-individual variability in both sprint performance and muscle activation was observed as external loads increased. These findings suggest that elite female sprinters maintain highly stable neuromuscular recruitment patterns, particularly in the quadriceps and hamstrings, when sprinting with external loads up to 15% BM, potentially reflecting a ceiling effect in their neuromuscular responsiveness. From a practical perspective, light-to-moderate RST may effectively stimulate posterior chain muscles without disrupting sprinting mechanics. Future longitudinal studies are warranted to explore the chronic adaptations to motorized RST and to determine whether the observed neuromuscular strategies are consistent across sexes.

## 1. Introduction

Sprinting is a complex neuromuscular activity that requires highly coordinated interactions between multiple muscle groups to generate propulsive force, maintain posture, and optimize movement efficiency. The application of external loads through sled towing, resistance bands, motorized resistance devices, or weighted vests has emerged as a widely adopted strategy to enhance sprint-specific strength and acceleration capabilities [1,2]. Such resisted sprint training (RST) alters both the mechanical and neural demands of sprinting, thereby influencing muscle activation profiles, sprint mechanics, and subsequent performance outcomes [3,4].

During maximal sprinting, the neuromuscular system operates near its physiological limits. Surface electromyography (EMG) enables the non-invasive analysis of neuromechanical behavior by capturing the amplitude and timing of electrical activity within active muscle groups [5]. Among these, the lower-limb muscles—particularly the quadriceps (vastus lateralis, rectus femoris), hamstrings (biceps femoris, semitendinosus), gluteal muscles (gluteus maximus, gluteus medius), and calf muscles (gastrocnemius, soleus), play central roles in force production across the different phases of sprinting [6,7]. Additionally, core muscles such as the abdominals and erector spinae stabilize the trunk and facilitate efficient force transfer, while upper limb muscles contribute to contralateral balance and arm swing dynamics [8,9].

Resisted sprinting modifies muscle recruitment strategies. Lighter loads (typically <10–20% of body mass [BM]) can elicit moderate increases in gluteal and hamstring activity without substantial disruption to sprinting mechanics [1]. In contrast, heavier loads (i.e., >20–30% BM) have been associated with altered horizontal force production, increased ground contact time, and changes in step length and trunk lean [3,10,11]. Some researchers argue that heavy RST when applied systematically can improve sprint performance without negatively affecting sprint kinematics, possibly due to adaptive neuromuscular adjustments [12,13]. However, discrepancies across studies may result from methodological variations, such as differences in sprint distances, resistance calibration, or temporal measurement windows [14,15]. Despite the growing body of evidence on RST, there remains a lack of high-resolution EMG data documenting how muscle activity patterns vary with load magnitude, especially under realistic sprint conditions using motorized resistance systems. Furthermore, limited research has evaluated post-load muscle activation during subsequent unresisted sprinting, which could reflect both fatigue and potentiation phenomena [16,17].

The present study aimed to examine how external loading modulates neuromuscular activity within RST sessions, as assessed via EMG, in elite female sprinters. Specifically, we investigated muscle activation patterns under three resisted sprint conditions (i.e., 5%, 10%, and 15% BM) [1] during maximal sprint efforts. Based on previous findings and physiological parameters [11,18,19], we hypothesized that increasing external resistance while sprinting would induce distinct, load-dependent responses in neuromuscular activation, particularly in posterior chain muscles such as the gluteus maximus and hamstrings.

## 2. Materials and Methods

### 2.1. Subjects

Due to the exceptional characteristics of this population, a convenience sample of ten elite female sprinters (age: 23 ± 2.8 years; BM: 58.3 ± 4.7 kg; height: 172.5 ± 4.5 cm) was recruited at the beginning of the 2025 indoor season. All participants were in good health and exhibited a high level of physical fitness following a standardized three-month preparatory period. Inclusion criteria required a minimum of five years of sprint-specific training and competitive experience at the national and international levels in the 60 m, 100 m, 200 m, and 400 m events. The sprinters were extensively familiarized with the resisted sprint protocols, which facilitated the successful completion of the testing procedures and confirmed that they were accustomed to the demands of the experimental design.

Of the ten participants, five were classified as specialists in the 100 m and 200 m events, while the remaining five were classified as 400 m specialists. Notably, a recent study involving five elite male and thirteen elite female 400 m sprinters demonstrated that the maximal sprinting speed (MSS) achieved over 60 m is the strongest predictor of 400 m performance (r = −0.94 between MSS and 400 m sprint time) [20]. This finding indicates that these sprinters share very similar sprint speed characteristics, thereby constituting a homogeneous sample. The study was approved by the Institutional Ethics Committee of the Jerzy Kukuczka Academy of Physical Education in Katowice (approval code: 3/2021 [17 June 2021]). All procedures were conducted in accordance with the Declaration of Helsinki, and written informed consent was obtained from all participants prior to data collection.

### 2.2. Study Design

Testing was conducted on a synthetic indoor track at our high-performance training center between 9:00 and 11:00 a.m. Environmental conditions (temperature ~20 °C, humidity ~50%) were monitored and kept stable across all sessions. Participants were instructed to refrain from caffeine consumption and strenuous activity for 24 h prior to testing. Total testing time per athlete was approximately 90 min. Each session commenced with a standardized warm-up, including 5 min of jogging, dynamic bodyweight exercises (e.g., push-ups, squats, split squats), and submaximal sprints over 5 m and 20 m. Sprint performance was assessed via two maximal 30 m sprints, initiated from a standing three-point stance after a verbal ‘go’ signal given by the same experienced examiner. Running times were recorded using dual-beam Witty Gate photocells (Microgate, Bolzano, Italy; measurement accuracy: 0.01 s). Athletes then performed six additional 30 m sprints under three resisted sprint conditions (i.e., 5%, 10%, and 15% BM), with two sprints per load. These loading ranges were selected based on the conditions that these young sprinters regularly use during their training routines, with the aim of reducing potential disruptions in sprint technique (i.e., sprint kinetics and kinematics). This approach helped to ensure that the testing conditions reflected their habitual training practices and allowed for ecologically valid comparisons [11,18,19]. The resisted sprints were completed in a randomized, counterbalanced order using a Latin square design, with each sprint separated by at least 5–6 min of passive recovery to mitigate order effects and minimize fatigue.

Resisted sprinting trials were performed using the SPRINT 1080 system (1080 Motion AB, Stockholm, Sweden), a motorized cable device that applies intelligent, adjustable resistance throughout each movement phase. The system enables high-frequency sampling (333 Hz) of speed (m/s), force (N), and power (W), with validated accuracy (±0.5% for speed and ≤4.8 N for force measures) [21,22]. Resistance was dynamically calibrated in real time based on measured BM (assessed to the nearest 0.1 kg on the day of testing). Rating of perceived exertion (RPE) was recorded immediately after each sprint to assess perceived effort and to verify that fatigue did not accumulate across trials. On average, RPE values ranged from 9 to 12 on the Borg 6–20 scale [5], indicating that the athletes perceived the efforts as light-to-somewhat hard and confirming that fatigue did not accumulate throughout the protocol. Intraclass correlation coefficients (ICC > 0.92) indicated high test–retest reliability across sprint conditions.

### 2.3. Procedures

#### Electromyographic (EMG) Data Collection

Surface EMG signals were recorded bilaterally using wearable Myontec MShorts (Myontec Ltd., Kuopio, Finland), which integrate textile-embedded bipolar electrodes into form-fitting shorts. These garments enable unobtrusive, real-time data acquisition during dynamic tasks. The electrodes were pre-moistened with tap water to reduce skin impedance and ensure optimal signal conductivity. Electrode placement was standardized and verified according to the SENIAM guidelines. Target muscles included the vastus lateralis, vastus medialis, and rectus femoris (quadriceps group); the biceps femoris, semitendinosus, and semimembranosus (hamstrings); and the gluteus maximus and gluteus medius (gluteal group). Reference electrodes were positioned along the lateral thigh (iliotibial tract) to minimize motion artifacts.

EMG signals were recorded continuously during sprint trials at a sampling rate of 1000 Hz and band-pass filtered between 50 and 200 Hz (analog filter, −3 dB). The raw signals were rectified and segmented into non-overlapping 100 ms epochs (10 Hz time resolution). These rectified epochs were averaged to obtain the average rectified value (ARV), the primary variable of interest, expressed in arbitrary units (AU), characteristic of the Myontec system. Data were stored in ASCII format using manufacturer-specific software and were subsequently processed using MATLAB R2024a (MathWorks Inc., Natick, MA, USA). Prior to analysis, all signals were visually inspected for noise and corrected where necessary to ensure high data quality.

### 2.4. Statistical Analysis

All data are presented as mean ± standard deviation (SD). Statistical analyses were conducted using IBM SPSS Statistics v27 (IBM Corp., Armonk, NY, USA) and R software R 4.4.0 (R Foundation for Statistical Computing, Vienna, Austria). Prior to inferential testing, assumptions of normality were verified using the Shapiro–Wilk test (*p* > 0.05 for all variables). Mauchly’s test of sphericity was used to assess the homogeneity of variances in repeated measures; if violated, the Greenhouse–Geisser correction was applied. A one-way repeated-measures (ANOVA) was employed to evaluate the effect of external load (i.e., baseline [unresisted sprints]; loads = 5%, 10%, and 15% BM) on 30 m sprint time and EMG-derived muscle activity. Significant effects were further explored using Tukey’s Honest Significant Difference (HSD) post hoc test. The significance level was set at α = 0.05. Effect sizes (ES) were calculated to supplement *p*-value interpretation. Eta-squared (η^2^) was reported for ANOVA results. Thresholds for interpretation were small (η^2^ = 0.01; d = 0.2), medium (η^2^ = 0.06; d = 0.5), and large (η^2^ = 0.14; d = 0.8), following conventional benchmarks.

## 3. Results

**Figure 1:**
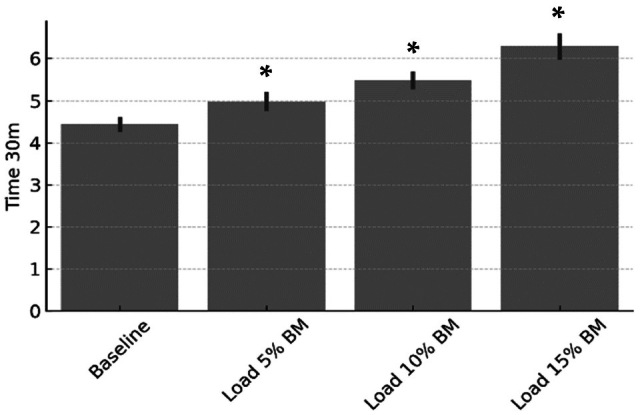
Thirty-meter sprint time (s) under different loading conditions. * *p* < 0.001 vs. Baseline.

The application of different external loads significantly affected sprint time and partially influenced neuromuscular activity during resisted sprinting. A one-way ANOVA revealed a significant main effect of condition on 30 m sprint time (F = 216.83, *p* < 0.001), indicating that increasing external resistance systematically slowed sprint performance (Table 1; Figure 1). Post hoc comparisons confirmed significant differences between all load conditions (5%, 10%, and 15% BM) and Baseline (*p* < 0.001), with the greatest performance decrement observed under the 15% BM load.

**Figure 2:**
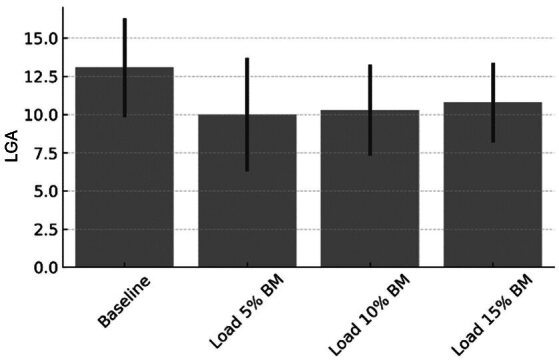
Left gluteus activation (LGA) [expressed in average rectified value, ARV (a.u.)] under different loading conditions. No significant differences were observed across conditions (*p* > 0.05); however, a trend toward increased activation was identified at heavier loads (*p* = 0.053).

In contrast, no significant differences were found in the activation of most individual muscle groups. Specifically, the left quadriceps (F = 0.31, *p* = 0.818), right quadriceps (F = 0.53, *p* = 0.663), right gluteus (F = 0.61, *p* = 0.614), and both left and right hamstrings did not show statistical variation across conditions. However, left gluteus activity approached significance (F = 2.74, *p* = 0.053), suggesting a potential trend toward increased activation at heavier loads (Figure 2).

SD analysis revealed increasing variance in sprint time with increasing external load. Under Baseline, SD for 30 m sprint time was minimal (±0.14 s), whereas under 15% load, inter-individual variability increased (±0.28 s), suggesting heterogeneous adaptation strategies among athletes. A similar pattern was observed in muscle activation variability, with the greatest dispersion found in the right gluteus and right hamstring under loaded conditions, further highlighting differential neuromuscular responses to the same external resistance.

## 4. Discussion

This study investigated the effects of different resistance loads (i.e., 5%, 10%, and 15% BM) on sprint speed and neuromuscular activation in elite female sprinters. Our results demonstrate that increasing external load during resisted sprinting systematically impairs sprint performance while partially modulating muscle activation patterns, particularly in the gluteal muscles. The substantial decline in sprint performance with progressive loading aligns with previous studies showing that increased horizontal resistance significantly alters sprint mechanics and force application strategies [10,13]. Notably, the extremely large ES observed for sprint time (η^2^ = 0.926) highlights the strong influence of external resistance on sprint speed, reinforcing the importance of precise and adequate load prescription to avoid excessive mechanical alterations and technical disruptions during RST [11,23,24].

Contrary to our initial hypothesis, most muscle groups did not exhibit significant changes in EMG activity across the different loading conditions. These findings partially contrast with previous research reporting load-dependent increases in posterior chain muscle activation within RST sessions [1]. It is important to note, however, that the loading conditions employed in our study may be considered relatively light (i.e., 5–15% BM), which, according to the same authors, should be prioritized to minimize—or even avoid—substantial disruptions in sprinting technique [2,11,23]. Given the limited scope for acute or chronic changes in this population (i.e., elite sprinters) [25,26], the trend toward increased left gluteus activity (*p* = 0.053; η^2^ ≈ 0.14) may reasonably be interpreted as meaningful, since the ES approaches the threshold for a large effect [27,28,29] despite the small sample size. This finding suggests that gluteal muscles may play a critical role in meeting the greater demands imposed by RST. In practical terms, this neuromuscular response underscores the complex and potentially individualized recruitment strategies employed by athletes when exposed to varying levels of external resistances, as reflected by the greater inter-individual variability observed under heavier loading conditions [30,31].

Interestingly, the lack of statistically significant changes in hamstring and quadriceps activation across loads may reflect the high technical proficiency of this elite sample of female sprinters. Given their extensive sprinting background and prior exposure to RST protocols, these athletes may have developed highly stable neuromuscular patterns, which could be further reinforced by the use of lighter loading conditions (i.e., ≤15% BM)—a strategy commonly adopted by Olympic sprint coaches [2,23]. This notion is supported by the consistently low variability observed in quadriceps activation across conditions and aligns with previous evidence suggesting that well-trained sprinters can preserve muscle coordination patterns and, consequently, running technique, even in the presence of mechanical perturbations [5,15].

In conclusion, the absence of significant alterations in hamstring and quadriceps activation across different external loads reflects the neuromuscular stability and technical consistency developed through years of specialized sprint training in this elite cohort of female sprinters. This finding suggests a potential ceiling effect in neuromuscular responsiveness among highly trained athletes—particularly competitive sprinters [25,32]—and reinforces the rationale for employing light loading conditions during RST (i.e., ≤10–15% BM) [23], a practice consistent with high-performance coaching strategies aimed at preserving sprint mechanics while eliciting targeted speed-strength adaptations [2,19].

Despite its uniqueness and relevance, this study has certain limitations. Its cross-sectional design precludes conclusions about long-term adaptations, and the small sample size—although common in research involving elite-level sprinters—may limit generalizability and reduce the robustness of the statistical analyses [26,33]. These constraints are inherent to research with highly specialized populations, where access to top-level athletes is limited and pre-established protocols cannot be modified or combined with non-standardized tests [34,35]. Future research should investigate the effects of systematically integrating light-to-moderate motorized RST into sprint training programs, with particular attention to acute and longitudinal changes and adaptations in sprint kinetics and kinematics (e.g., step and stride length, flight time, ground contact time, ground reaction forces, vertical and horizontal impulse), neuromuscular recruitment strategies, and potential reductions in injury risk. Furthermore, studies involving male and female sprinters from different performance levels are warranted to determine whether the neuromuscular patterns and changes observed here are consistent across sexes and competitive standards.

## 5. Conclusions

From a practical standpoint, these results support the use of RST with light external loads (i.e., ≤10–15% BM) as an effective strategy to modulate posterior chain muscle activation without compromising sprinting technique. Given the observed neuromuscular consistency in quadriceps and hamstring activation across the analyzed loading conditions (i.e., 5%, 10%, and 15% BM), practitioners working with highly trained female sprinters may employ light-loaded resisted sprints to reinforce specific and stable recruitment patterns and address individual needs related to strength and speed development. Moreover, the load-sensitive behavior of gluteal and hamstring muscles highlights the potential of RST to selectively target posterior chain activation, providing an applied and safe approach to enhance speed-strength qualities in sprinters or athletes from other sports. Nevertheless, it is worth noting that excessively heavy loads may induce substantial alterations and disruptions in muscle activation patterns [19,23], which, in addition to increasing fatigue, could compromise sprinting technique [2,19,23]. At the same time, it remains uncertain whether such loading conditions might elicit distinct EMG responses in elite sprinters over prolonged exposure to RST [23].

## Figures and Tables

**Table 1:** Descriptive statistics (mean ± SD) for sprint time and muscle activity variables under different loading conditions.

Group	Time 30 m (s)	LQ	RQ	LG	RG	LH	RH
Baseline	4.44 ± 0.14	18.1 ± 3.7	18.4 ± 3.5	13.1 ± 3.3	16.9 ± 5.4	17.9 ± 3.2	15.6 ± 2.2
Load 5% BM	4.98 ± 0.19 *	19.4 ± 4.1	20.0 ± 4.1	10.0 ± 3.7	14.4 ± 6.9	19.1 ± 3.6	17.1 ± 2.6
Load 10% BM	5.48 ± 0.17 *	19.2 ± 3.3	18.9 ± 2.4	10.3 ± 3.0	14.0 ± 5.7	19.1 ± 3.3	18.3 ± 3.4
Load 15% BM	6.29 ± 0.28 *	18.7 ± 3.6	18.8 ± 3.6	10.8 ± 2.6	15.1 ± 6.2	18.4 ± 3.5	18.2 ± 3.4

Note: BM: body mass; LQ: left quadriceps; RQ: right quadriceps; LG: left gluteus; RG: right gluteus; LH: left hamstrings; RH: right hamstrings. * *p* < 0.001 vs. Baseline.

## Data Availability

The data that support the findings of this study are available from the corresponding author upon reasonable request. The data are not publicly available due to restrictions imposed by the Polish Olympic Committee (Polski Komitet Olimpijski) for studies involving athletes from the National Team.
